# ICD-9 Codes and Surveillance for *Clostridium difficile*–associated Disease

**DOI:** 10.3201/eid1210.060016

**Published:** 2006-10

**Authors:** Erik R. Dubberke, Kimberly A. Reske, L. Clifford McDonald, Victoria J. Fraser

**Affiliations:** * Washington University in St. Louis–School of Medicine, Saint Louis, Missouri, USA;; † Centers for Disease Control and Prevention, Atlanta, Georgia, USA;; ‡ Barnes-Jewish Hospital, Saint Louis, Missouri, USA

**Keywords:** *Clostridium difficile*, International Classification of Diseases, hospital infections, dispatch

## Abstract

We conducted a retrospective cohort study to compare *Clostridium difficile*–associated disease rates determined by *C. difficile*–toxin assays and International Classification of Diseases, 9th Revision (ICD-9) codes. The correlation between toxin assay results and ICD-9 codes was good (κ = 0.72, p<0.01). The sensitivity of the ICD-9 codes was 78% and the specificity was 99.7%.

*Clostridium difficile*–associated disease (CDAD) is the most common infectious cause of healthcare-associated diarrhea (*1*). Recent studies suggest both the incidence and severity of CDAD may be increasing (*2**–**9*), but no national surveillance system exists to track CDAD rates. Some studies have used International Classification of Diseases, 9th Revision (ICD-9) codes of hospital discharges to study CDAD rates (*4**,**10**,**11*). The validity of this method has not been studied. We compared CDAD rates determined by ICD-9 codes to rates determined by *C. difficile*–toxin assays at a tertiary-care hospital to determine the sensitivity and specificity of ICD-9 code–based CDAD surveillance.

## The Study

Data were collected electronically on a retrospective cohort of patients admitted to Barnes-Jewish Hospital in Saint Louis from January 1, 2003, through December 31, 2003. Patients who had only 1 admission of <48 hours and neonates were excluded. Electronic charts were reviewed for patients who had a positive *C. difficile*–toxin assay or the ICD-9 code indicating *C. difficile*–associated disease (008.45) (Appendix) .

A case of CDAD was defined as a patient with a positive *C. difficile*–toxin assay (Tech Laboratory *C. difficile* tox A/B II toxin assay [Tech Laboratory, Blacksburg, VA, USA]) or pseudomembranes seen on colonoscopy. Because the hospital laboratory performs a *C. difficile*–toxin assay only on unformed stool samples and stool toxin testing is ordered based on clinical suspicion of CDAD, all patients with a positive toxin assay were considered CDAD case-patients.

Data were analyzed with SPSS 12.0 for Windows (SPSS, Inc., Chicago, IL, USA). Statistical analyses included κ, χ^2^, and Mann-Whitney U tests. A 2-sided p value of 0.05 was considered significant. This study was approved by the Washington University Human Studies Committee.

**Figure 1:**
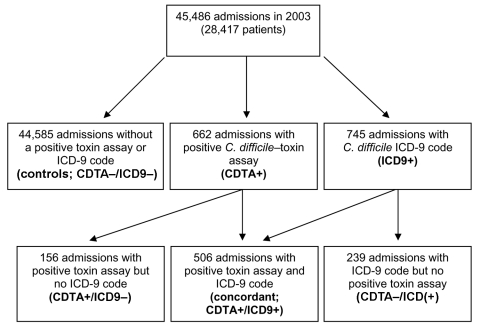
Flowchart of admission groups.

**Figure 2:**
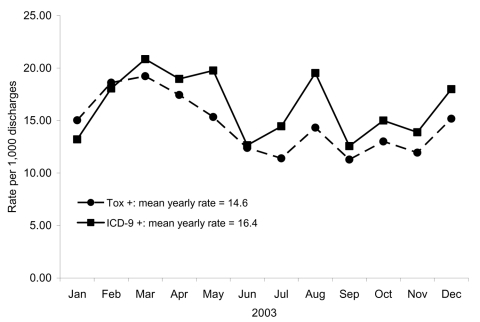
Monthly rates of *Clostridium difficile*–associated disease by diagnosis type.

A total of 45,486 admissions among 28,417 unique patients were included in the analysis (Figure 1). A *C. difficile*–toxin assay was ordered during hospitalization for 3,630 (8%) of these admissions. Toxin assays were positive (CDTA+) in 662 (18%) admissions. The *C. difficile* ICD-9 code was assigned to 745 admissions (ICD9+). The breakdown of admissions, according to toxin assay and ICD-9 status, was as follows: 506 had both a positive toxin assay and received the ICD-9 code (concordant; CDTA+/ICD9+), 156 had a positive toxin assay but no ICD-9 code (CDTA+/ICD9–), and 239 received the ICD-9 code but did not have a positive toxin assay (CDTA–/ICD9+) (Figure 1). The concordance between toxin assays and ICD-9 codes was good (κ = 0.72, p<0.01). The overall mean CDAD rate by ICD-9 codes (16.4/1,000 admissions) was significantly higher then the mean rate by toxin assays (14.6/1,000 admissions) (Figure 2; rate ratio [RR] 1.13, 95% confidence interval [CI] 1.01–1.25).

The median number of days from admission to stool collection was greater in admissions with a positive toxin assay but no ICD-9 code (CDTA+/ICD9–) than in concordant (CDTA+/ICD9+) admissions (6.0 days vs 3.0 days, p<0.01) (Table 1). The first positive stool sample was collected within 48 hours of discharge for 68 (44%) of admissions with a positive toxin assay only (CDTA+/ICD9–) admissions, compared with 72 (14%) of concordant (CDTA+/ICD9+) admissions (p<0.01).

**Table 1:** Demographic characteristics of study population by *Clostridium difficile*–toxin assay (CDTA) and ICD-9 status*

Characteristic	Controls, n = 44,585 (%)	CDTA+/ICD-9–, n = 156 (%)	CDTA+/ICD-9+, n = 506 (%)	CDTA–/ICD-9+, n = 239 (%)
Age (median y)	55	64	67	66
Length of hospitalization (median d)	4	13	12	6
Female	25,869 (58)	68 (44)	267 (53)	158 (66)
White	28,071 (63)	110 (71)	347 (69)	170 (71)
Time from admission to stool collection (median d)	NA	6.0	3.0	NA
First positive stool collected within 48 h of discharge	NA	68 (44)	72 (14)	NA

*NA. not available.

Upon chart review, documentation of a previous history of CDAD was evident in 142 (59%) of the ICD-9 code only (CDTA–/ICD9+) admissions. A *C. difficile*–toxin assay had been ordered in 137 (57%) of all ICD-9 code only (CDTA-/ICD9+) admissions. One-hundred thirty (54%) had at least 1 stool negative for *C. difficile* toxin.

Overall, 92% of patients with positive toxin assay (CDTA+) and 90% of patients with ICD-9 code only (CDTA–/ICD9+) received antimicrobial drug treatment for CDAD (Table 2). Metronidazole was prescribed in 187 (78%) of the ICD-9 code only (CDTA–/ICD9+) admissions, compared with 591 (89%) of patients with a positive toxin assay (CDTA+) (p<0.01). For 75 (31%) of the ICD-9 code only (CDTA–/ICD9+) admissions, oral vancomycin was prescribed, compared with 130 (20%) of patients with a positive toxin assay (CDTA+) (p<0.01).

**Table 2:** Comparison of antimicrobial treatment for *Clostridium difficile*–associated disease among patient admissions with a positive *C. difficile*–toxin assay (CDTA+) and patients without a positive toxin assay but with ICD-9 code for *C. difficile* disease (CDTA–/ICD9+) (categories not mutually exclusive)

Treatment	CDTA+, n = 662 (%)	CDTA–/ICD9+, n= 239 (%)	Odds ratio	p value
Any treatment for CDAD	607 (92)	214 (90)	0.78	0.35
Metronidazole	591 (89)	187 (78)	0.43	<0.01
Oral vancomycin	130 (20)	75 (31)	1.87	<0.01
Oral vancomycin and metronidazole	114 (17)	48 (20)	1.21	0.32

Thirty-four cases of CDAD were missed by *C. difficile*–toxin assay results and subsequently identified through chart review (3 missed positive toxins, 26 CDAD patients transferred from other facilities, 3 positive outpatient toxin assays, and 2 diagnosed by colonoscopy), which brought the total number of cases with positive CDAD diagnostics to 696. ICD-9 codes correctly identified 540 of these cases and correctly classified 44,741 admissions as non-CDAD admissions (sensitivity 78%, specificity 99.7%). When the CDAD rate by toxin assays was adjusted for the additional cases, the adjusted CDAD rate was 15.3/1,000 admissions. This rate was not significantly different from the unadjusted CDAD rate by toxin assay results (RR 0.95, 95% CI 0.86–1.06) or the rate by ICD-9 codes alone (RR 1.07, 95% CI 0.97–1.19).

## Conclusions

Overall, there was good correlation between *C. difficile*–toxin assay results and ICD-9 codes. Initially, the CDAD rate by ICD-9 codes appeared higher than the rate by toxin assays. However, once the additional CDAD cases identified through chart review were added, this difference was not significant.

Admissions with only a positive *C. difficile*–toxin assay and no *C. difficile* ICD-9 code (CDTA+/ICD9–) were more likely than concordant (CDTA+/ICD9+) admissions to have their first positive toxin assay within 48 hours of discharge. For these admissions, toxin assay results may not yet have been back at the time of discharge or CDAD may not have been considered a primary diagnosis by the physician and therefore not captured by the medical coders.

Antimicrobial drug treatment patterns suggest ICD-9 code only (CDTA–/ICD9+) admissions were patients who were more likely to have a history of CDAD. Metronidazole is first-line therapy for CDAD at our institution. Oral vancomycin is reserved for recurrent or severe cases. The observation that more ICD-9 code only (CDTA–/ICD9+) patients received oral vancomycin indicates that recurrent CDAD may have been suspected in these patients. In these patients, CDAD appears to have been diagnosed on the basis of the patient's history and symptoms instead of by a positive *C. difficile*–toxin assay. This pattern has been previously reported (*12*).

True CDAD cases may have been misclassified among the controls. A patient who did not have a positive *C. difficile*–toxin assay, who was not assigned the CDAD ICD-9 code, and whose diagnosis was made by colonoscopy would have been missed. However, misclassification is unlikely for two reasons. First, after charts were reviewed, only 2 additional patients were identified whose diagnosis was made by colonoscopy alone. Second, the detection of CDAD cases transferred from other institutions indicates that CDAD cases diagnosed by methods other than the toxin assays are being captured by ICD-9 codes.

Use of ICD-9 codes to study CDAD rates has advantages and disadvantages. ICD-9 codes are readily available from billing databases. In the absence of a national surveillance system for CDAD, ICD-9 codes provide a standard method for determining CDAD rates that can be used at all types of hospitals. However, because ICD-9 codes are assigned at discharge and not on the date of diagnosis, determining which cases are hospital acquired and which are community acquired is difficult. Also, ICD-9 codes are assigned by medical coders, who may not be able to accurately identify a patient's principal diagnoses as well as a physician or medical professional. Despite these limitations, ICD-9 codes can likely be used to identify CDAD cases and track CDAD rates when *C. difficile*–toxin assay results are not available.

## Appendix

### Details on ICD-9 Codes

The International Classification of Diseases, 9th Revision, Clinical Modification (ICD-9) code used in this study was 008.45, "intestinal infection due to *Clostridium difficile*," and is the only ICD-9 code related to CDAD. To apply this code, medical coders must have documentation in a patient's medical record by the treating medical providers that a patient's gastroenteritis or colitis is due to *C. difficile*. Positive laboratory tests alone are not sufficient to warrant application of the code. At our institution, ICD-9 coding occurs, on average, 5–7 days after a patient is discharged from the hospital.

The ICD-9 system of classifying hospital discharge diagnoses is used throughout the United States. The definition for the code 008.45 is consistent between hospitals, although individual coding practices may vary. Although ICD-9 codes have limitations, they are readily available from administrative databases and have been used frequently to identify diagnoses and classify comorbidities (1).

A move to the International Classification of Diseases, 10^th^ Revision, Clinical Modification (ICD-10) system is anticipated for US hospitals but the exact time of this transition is not yet known. The ICD-10 system does include a code for CDAD (A04.7, Enterocolitis due to *C. difficile*), so the ICD-based system presented here could be modified to be used with the updated coding system.

### Appendix Reference

1. Klabunde CN, Warren JL, Legler JM. Assessing comorbidity using claims data: an overview. Med Care. 2002;40(8 Suppl):IV-25–35.
